# Altered effective connectivity in migraine patients during emotional stimuli: a multi-frequency magnetoencephalography study

**DOI:** 10.1186/s10194-021-01379-4

**Published:** 2022-01-15

**Authors:** Jing Ren, Qun Yao, Minjie Tian, Feng Li, Yueqiu Chen, Qiqi Chen, Jing Xiang, Jingping Shi

**Affiliations:** 1 Department of Neurology, The Affiliated Brain Hospital of Nanjing Medical University, Nanjing, 210029 Jiangsu China; 2 MEG Center, The Affiliated Brain Hospital of Nanjing Medical University, Nanjing, 210029 Jiangsu China; 3 MEG Center, Division of Neurology, Cincinnati Children’s Hospital Medical Center, Cincinnati, OH 45220 USA

**Keywords:** Migraine, Magnetoencephalography, Multi-frequency, Emotional stimuli, Effective connectivity

## Abstract

**Background:**

Migraine is a common and disabling primary headache, which is associated with a wide range of psychiatric comorbidities. However, the mechanisms of emotion processing in migraine are not fully understood yet. The present study aimed to investigate the neural network during neutral, positive, and negative emotional stimuli in the migraine patients.

**Methods:**

A total of 24 migraine patients and 24 age- and sex-matching healthy controls were enrolled in this study. Neuromagnetic brain activity was recorded using a whole-head magnetoencephalography (MEG) system upon exposure to human facial expression stimuli. MEG data were analyzed in multi-frequency ranges from 1 to 100 Hz.

**Results:**

The migraine patients exhibited a significant enhancement in the effective connectivity from the prefrontal lobe to the temporal cortex during the negative emotional stimuli in the gamma frequency (30–90 Hz). Graph theory analysis revealed that the migraine patients had an increased degree and clustering coefficient of connectivity in the delta frequency range (1–4 Hz) upon exposure to positive emotional stimuli and an increased degree of connectivity in the delta frequency range (1–4 Hz) upon exposure to negative emotional stimuli. Clinical correlation analysis showed that the history, attack frequency, duration, and neuropsychological scales of the migraine patients had a negative correlation with the network parameters in certain frequency ranges.

**Conclusions:**

The results suggested that the individuals with migraine showed deviant effective connectivity in viewing the human facial expressions in multi-frequencies. The prefrontal-temporal pathway might be related to the altered negative emotional modulation in migraine. These findings suggested that migraine might be characterized by more universal altered cerebral processing of negative stimuli. Since the significant result in this study was frequency-specific, more independent replicative studies are needed to confirm these results, and to elucidate the neurocircuitry underlying the association between migraine and emotional conditions.

## Introduction

Migraine is a common primary headache characterized by moderate to severe pain and accompanied by vomiting, nausea, photophobia, and phonophobia [1]. Due to its high prevalence and social burden, migraine has emerged as public health concern worldwide [2]. However, the neuropathological mechanisms and treatment strategies for migraine are not known yet. Therefore, it is imperative to explore the pathological mechanism of migraine for developing more effective therapeutic strategies.

Pain is defined as an unpleasant sensory and emotional experience [3]. The correlations among different emotional factors and headache symptoms are complex and bidirectional [4–7]. Migraine have a high prevalence of psychiatric comorbidities, such as depression, anxiety, and post-traumatic stress disorder [5, 8, 9]. Moreover, stress is reported as a prevalent trigger for migraine and might also mediate the association between migraine and other psychiatric comorbidities [10, 11]. The biological mechanisms of complex correlations among psychiatric comorbidities have not been explained yet.

Neuroimaging technology provides a better understanding of the neural dysfunction in migraine. Studies based on neuroimaging have demonstrated abnormal brain regions activation upon exposure to sensory stimuli, absence of the normal habituating response between the attacks, and the atypical functional connectivity of sensory processing brain areas [12]. Furthermore, previous studies have also reported that the migraine patients have overlapped network, involving pain, emotion, and migraine [13–17]. As mentioned before, pain is a negative emotional experience. Some studies suggest that the migraine patients have abnormal processing of multiple sensory information during and between the headache attacks, including visual, auditory, and gustatory information [18, 19]. These sensory stimuli might also involve a significant negative emotional component. Therefore, the abnormal processing of multiple stimuli might be induced by an unpleasant emotion rather than specific sensory stimulus.

Human facial expressions, as a type of emotional stimuli, have been used to investigate the processing of central nervous system. In an event-related potential study, migraine group displayed a significant increase in N170 amplitude toward angry faces as compared to neutral faces [20]. This phenomenon can be interpreted as a differential process in emotional facial expression preferentially and intensively towards unpleasant stimuli. Besides, previous studies have shown that the migraine patients had altered brain activation under negative emotion stimuli using functional magnetic resonance imaging MRI (fMRI) and electroencephalogram (EEG) [20–22]. In these studies, the migraine patients had stronger activation of their visual cortex, cerebellum, amygdala, and posterior cingulate gyrus as compared to those of healthy volunteers. Furthermore, alterations in the pain-related connectivity in migraine, investigated using fMRI, EEG, and magnetoencephalography (MEG), have recently been reviewed [23], which showed that brain regions had atypical connectivity involved in the pathophysiological mechanisms of migraine. Many brain areas related to the processing of affective emotion were also included, such as amygdala, temporal lobe, and prefrontal lobe.

MEG, a non-invasive device with multiple frequency bands, has been used to investigate neurological disorders. Equipped with a high temporal resolution, MEG can detect a subtle difference in neuronal activity [24]. A previous study of migraine, using MEG, revealed an abnormal connectivity network at rest and under somatosensory stimulation as compared to controls [25, 26]. To the best of our knowledge, MEG has not been used to examine the neural networks in response to facial expression stimuli in the migraine patients.

The present study was designed to discover the whole-brain effective connectivity (EC) towards human emotional facial expression in the migraine patients during headache-free phase using MEG. Negative, positive, neutral facial expressions were examined and neuromagnetic signals from 1 to 100 Hz band were recorded. Furthermore, the impacts of migraine characteristics were also evaluated. We hypothesis a significantly enhanced EC was observed in the brain regions involved in emotion and pain processing in response to negative emotional stimuli in the migraine group as compared to the controls. Moreover, the disruptions of whole-brain EC might be associated with the neuropsychological scores of migraine patients.

## Methods

### Participants

A total of 24 migraine patients without aura (18 females, mean age = 29.25, SD = 9.32) and 24 controls (16 females, mean age = 29.71 years, SD = 7.46) were included in this study. All the patients were recruited from the outpatient department of Nanjing Brain Hospital, Nanjing, China. The study was approved by the medical ethics committee of Nanjing Brain Hospital. Each subject provided a written informed consent. The inclusion criteria for the migraine patients were based on the International Classification of Headache Disorders, 3rd edition (ICHD-IIIbeta) of 2013 (Headache Classification Subcommittee of the International Headache Society, 2013). Exclusion criteria for the migraine group patients included: 1) diagnosis with other neurological diseases in clinical interviews and structural MRI; 2) existence of psychiatric disease, as defined by the the Diagnostic and Statistical Manual of Mental Disorders, Fifth Edition(DSM-V); and 3) use of prophylactic drugs within 1 week before the test. Gender- and age-matching healthy controls were recruited through a poster on the outpatient department of Nanjing Brain Hospital. Most of the healthy controls were college students and the relatives of patients. Healthy controls never reported any migraine history or other types of headache attacks or any psychiatric and neurological diseases in the past years. The common exclusion criteria for all the participants included: 1) history of ferromagnetic implants; 2) history of brain damage; and 3) lack of ability to keep still. All the subjects were right-handed. The recruited migraine patients had no headache attacks 72 h before and 24 h after MEG recording. The features of migraine clinical assessment included headache history, headache frequency, duration of last headache attack, accompanying symptoms, and pain intensity (visual analogue scale, VAS) were collected. The Hamilton Anxiety rating scale (HAM-A) and Hamilton Depression rating scale (HAM-D) were used to evaluate the subjects’ anxiety and depression symptoms.

### Stimuli and procedure

**Fig. 1:**
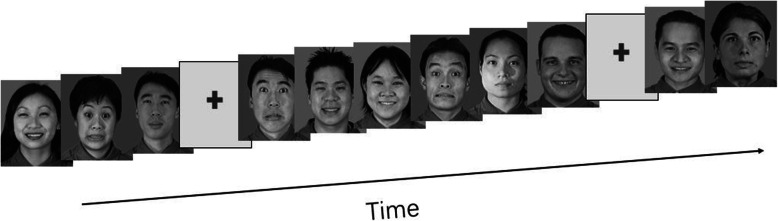
Emotional task paradigm

Human facial expression images were used as emotional stimuli in this study. The images were categorized into negative, positive, and neutral faces. The stimuli consisted of 180 grey-scale images taken from the NimStim Set of Facial Expressions [27] and the Montreal Set of Facial Displays of Emotion [28]. Additionally, cross fixation targets were randomly inserted into 18 images to ensure the focus of each subject on the images. The images were displayed in a video using BrainX software [29]. The images were randomly presented for 500 ms followed by 2000–2500 ms of inter-trial intervals. All the images were set in the center of a black background. The experimental paradigm is presented in Fig. 1.

### MEG signals recording

The MEG data were collected in a magnetic shielding room in the MEG center at Nanjing Brain Hospital using a whole-head 275-Channel MEG system (VSM Medical Technology Company, Canada). All the patients had no migraine attacks for 72 h before MEG, during, or 24 h after MEG sampling. Before MEG recording, three electromagnetic coils were attached to the reference landmarks on the left and right pre-auricular points and nasion of each participant in order to check the head position. All the subjects were instructed to lay comfortably in a positive supine gesture, remain still, and avoid moving heads. During data scanning, each patient kept gazing at the screen and pressed a button attached with their right hand when they saw a fixation cross in the video. The MEG sample frequency was 6000 Hz. Head positions were measured at the beginning and end of the sampling. If the head moved beyond 5 mm, the data were excluded and sampled again. All the recorded data were subjected to a noise cancellation of third-order gradients.

### MRI scanning

The structural T1 images of all the subjects were collected using a 1.5 T MRI (Singa, GE, USA). Three markers were placed on the nasion and bilateral pre-auricular points to identify the positions of the three coils during the MEG data scanning to facilitate the co-registration of MRI and MEG data. All the anatomical landmarks, which were subsequently digitized in the MEG scanning, were identified in MRI.

### Data processing

**Fig. 2:**
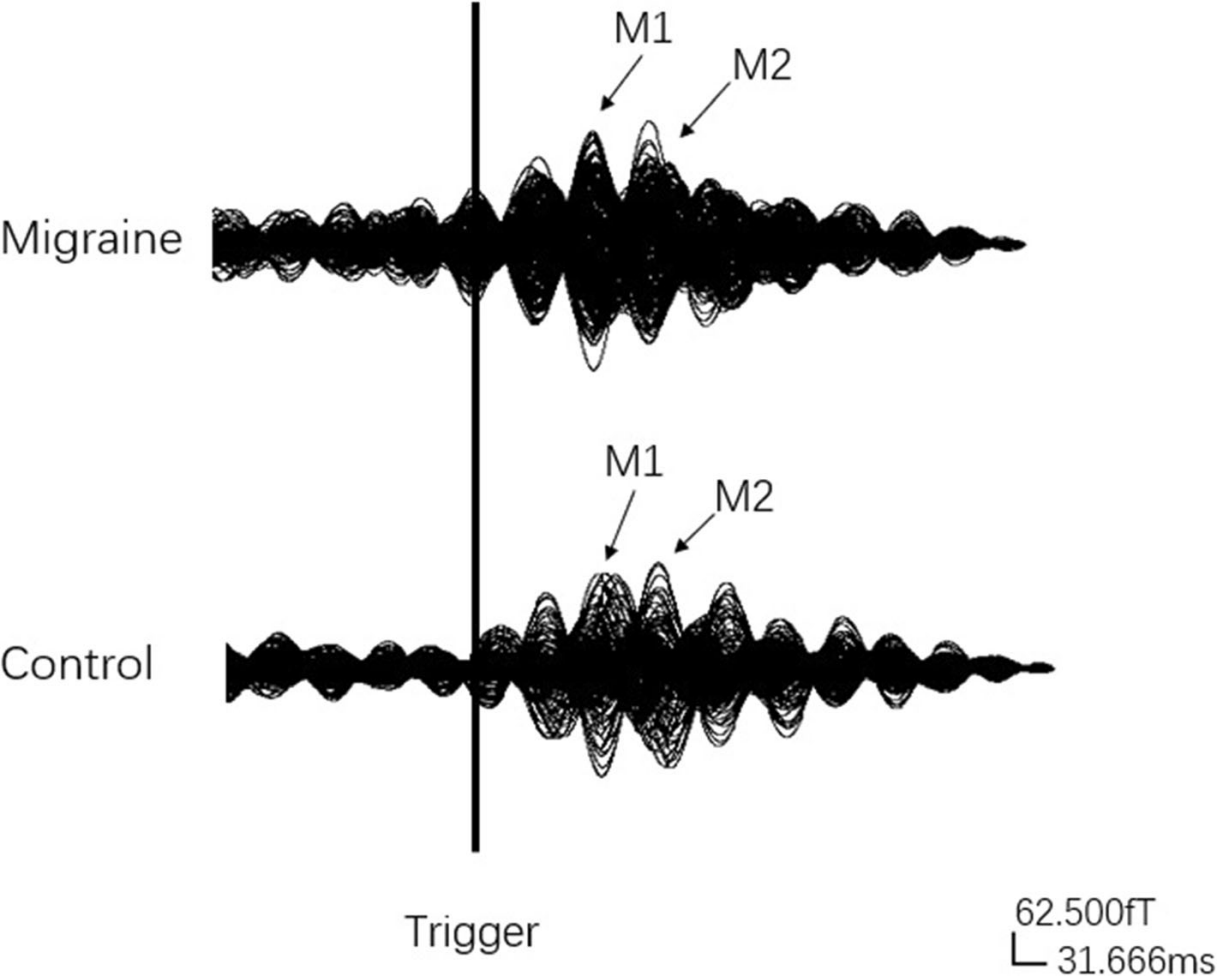
Magnetoencephalography (MEG) waveforms, showing neuromagnetic activation upon exposure to emotional stimuli in a migraine subject and a healthy subject in the 8–12 Hz frequency range. “M1” and “M2” were the two main components evoked by human facial expressions. The “Trigger” indicates the start of emotional stimuli

The averaged data without noise and other artifacts were marked as “clean data”, preprocessed by removing the direct current offset, and two main neuromagnetic components were obtained. After triggering from MEG data, 90-180 ms was selected as a time window for analysis. All the data were filtered using band-pass filters at pre-defined bandwidths of delta (1–4 Hz), theta (4–8 Hz), alpha (8–12 Hz), beta (13–30 Hz), and gamma (30–90 Hz), and a 50 Hz notch filter was used to eliminate power-line noise. All the data proceeded to additional analyses in these bands separately. A sample data is shown in Fig. 2.

Based on previous studies, the neural network was investigated at the source level [26]. Granger causality (GC) and covariance analysis were used to estimate the EC. In order to analyze the EC network, a significant neuromagnetic activity was selected using a real-time source imaging and defined as the volumetric source activity (or virtual sensor waveform) at each time point; this was specifically developed and optimized to analyze the activities at multiple frequency bands [30, 31]. The source activity was computed using a two-step beamformed method. The detailed method has been described in previous studies [24, 30]. Whole brain was scanned at 6-mm resolution (around 17,160 voxels/source) in this study. The correlations among all virtual sensor signals in the time-windows corresponding to the event-related magnetic fields were analyzed to estimate the global connectivity [24, 32]. Next, we statistically analyzed the correlation of two virtual sensor signals between two source pairs by calculating the correlation coefficient based on Eq. (1).

1 $$ R\left({X}_a,{X}_b\right)=\frac{C\left({X}_a,{X}_b\right)}{S_{X_a}{S}_{X_b}} $$

where R (X_a_, X_b_) represents the correlation between two source pairs at two positions (“a” and “b”), Xa and Xb indicate the MEG signals of two paired sources, which were used to calculate connections, and C (Xa, Xb) and SxaSxb represent the mean and standard deviation of the signals in the two sources, respectively. Moreover, in order to reduce bias, every possible connection was calculated for each dual-source pair at the source level. Notably, any two voxels less than 10 mm was recognized as one voxel.

Similar to recent studies, a multivariate GC was used to analyze the directivity of connections [33, 34]. It was interpreted that if one source activity could predict another source activity in a few milliseconds, the two sources were defined as connected. Otherwise, the two sources were not connected. Then, the neuromagnetic networks at the source level were overlapped onto the structural MRIs of individual subjects. The EC was visualized based on the magnetic source imaging in three views (axial, coronal, and sagittal) in order to analyze the excitatory and inhibitory connections, which were shown in red and blue. An excitatory connection represented a positive connection, where the amplitude of the signals in a source pair was positively correlated, while an inhibitory connection represented a negative connection, where the amplitude of the signals in two connected sources was negatively correlated [33]. Yellow point indicated the node drive to other nodes, while pink point indicated that the node was driven by other nodes.

Furthermore, graph theory analysis was also performed to quantify the characteristics of neural networks at the source level. A graph is a mathematical representation of a network and consists of a set of nodes and edges. In order to exactly define and compare the global EC network properties, the degree (D), strength (S), characteristic path length (L), and clustering coefficient (C) were calculated for each source pairs. The D of a node, which was often used to quantify centrality, indicated the number of connections between the node and others. L represented the number of edges in a network [35]. S was defined as the strength of all possible links to all sources. C referred to the probability that the neighbors of a node were also connected and considered to be a measure of the local connectivity in a graph. A threshold was set as a checkpoint to guarantee the data quality. All the networks with EC values above the threshold were shown. In order to define the threshold, the t values for all source pairs were calculated using Eq. (2).

2 $$ {T}_P=R\sqrt{\frac{K-2}{1-{R}^2}} $$

where Tp indicated the t value of a correlation, R indicated the correlation of a source pair, and K represented the number of data points in a connection. The Tp value had a corresponding *P*-value of < 0.01 as threshold for obtaining the EC network. All the algorithms were performed using an MEG Processor software (Cincinnati, OH, USA).

### Statistical analyses

The software package SPSS version 19.0 (IBM, Inc.) was used to perform statistical analyses. The EC network patterns in the migraine patients and controls were visually inspected and analyzed using Fisher’s exact test. A two-tailed student’s *t*-test was applied to assess the network properties between the migraine patients and controls. The correlation analyses were estimated using Spearman’s correlation coefficients. *P* < 0.05 was set as a threshold for the significance of difference, and Bonferroni correction was used to correct the multiple comparisons (for five frequency band *P* < 0.01 (0.05/5), for up to the four parameters of graph theory for each frequency band, *P* < 0.0025 (0.05/5 × 4 = 0.05/20 = 0.0025). False discovery rate (FDR) was applied to reduce the type I errors [36, 37].

## Results

### Demographics and clinical characteristics

As compared to the healthy controls, the migraine patients showed no differences in age, and gender, as listed in Table 1. In addition, the migraine patients presented a detailed score on the headache and neuropsychology scale.

**Table 1:** Clinical features and neuropsychological evaluation of subjects

Parameter	Migraine	Control
Gender (women/men)	18F/6M	16F/8M
Age (years)	29.25 ± 9.32	29.71 ± 7.46
Disease history (years)	11.17 ± 6.62	N/A
Frequency (times/month)	4.67 ± 4.40	N/A
Durations of migraine attacks (hours)	20.76 ± 17.71	N/A
Accompanied symptoms with attack N		
Phonophobia	20	N/A
Photophobia	18	N/A
Nausea/Vomiting	17	N/A
Locus of headache N		
Bilateral	7	N/A
Unilateral	17	N/A
Pain type (number of subjects)		
Throbbing	18	N/A
Pressure	3	N/A
Sharp	2	N/A
Stabbing	1	N/A
VAS of attack intensity (0–10)	7.42 ± 1.44	N/A
HAM-A Rating	9.67 ± 4.63	1.00 ± 0.65
HAM-D Rating	10.33 ± 5.11	1.58 ± 0.78

*N* number, *VAS* visual analog scale, HAM-A The Hamilton Anxiety Scale, HAM-D Hamilton Depression Scale

### Network pattern

It was found that the majority of the migraine patients (19/24) showed an increased EC from the prefrontal cortex (PFC) to the temporal lobe (TL) during negative emotional stimuli in the gamma band (30-90 Hz) as compared to the controls (8/24). The controls showed dominant anterior-posterior connections (*P* = 0.003 < 0.05, after Bonferroni correction). No significant differences were observed between the two groups upon exposure to positive and neutral stimuli.

**Fig. 3:**
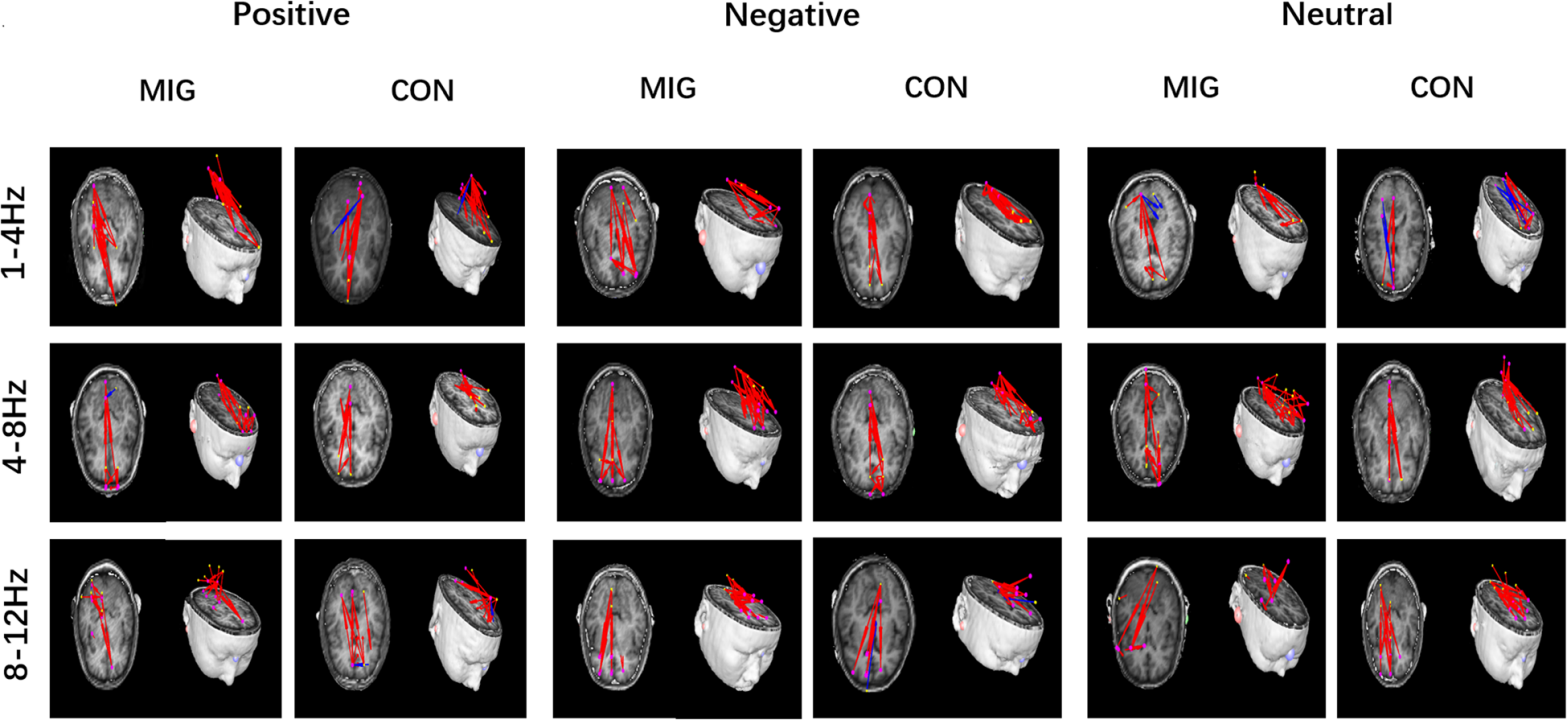
Typical predominant EC networks in the 1–12 Hz frequency range in migraine patients and controls visualized from the axial (left column) and lateral (right column) views. No significant difference was observed between the two groups when exposed to emotional stimuli. (Colored figure online)

**Fig. 4:**
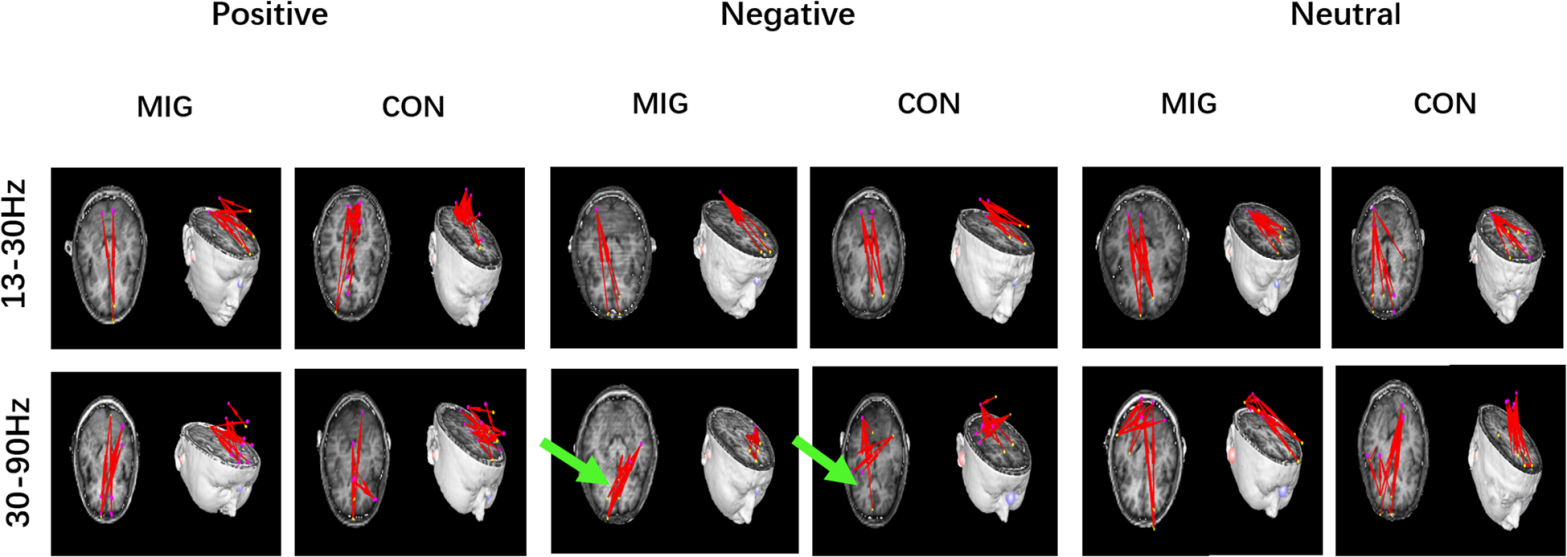
Typical EC network patterns in the 13–90 Hz frequency range in migraine patients and controls in response to positive, negative, and neutral human expressions, respectively, visualized from the lateral (left column) and axial (right column) views. The migraine patients showed a significantly altered pattern of EC network at 30–90 Hz frequency range in viewing the negative emotional stimuli as compared to the controls, showing more connections from the prefrontal lobe to the temporal lobe, which are indicated by green arrows. No significant differences were observed between the two groups exposure to positive and neutral stimuli. (Colored figure online)

In some frequency ranges (1–4 Hz, 4–8 Hz, 8–12 Hz, and 13–30 Hz), although their topographic patterns vary between them, the migraine patients and controls mostly showed excitatory connections between the sources in frontal cortex and occipital cortex. No significant difference was observed between the patients and controls under positive, neutral, and negative emotional stimuli in these frequency ranges. The details are shown in Figs. 3 and 4.

### Graph theory

The analysis of graph theory showed a significant difference in the graph theory parameters of the patients as compared to those of the controls.

**Fig. 5:**
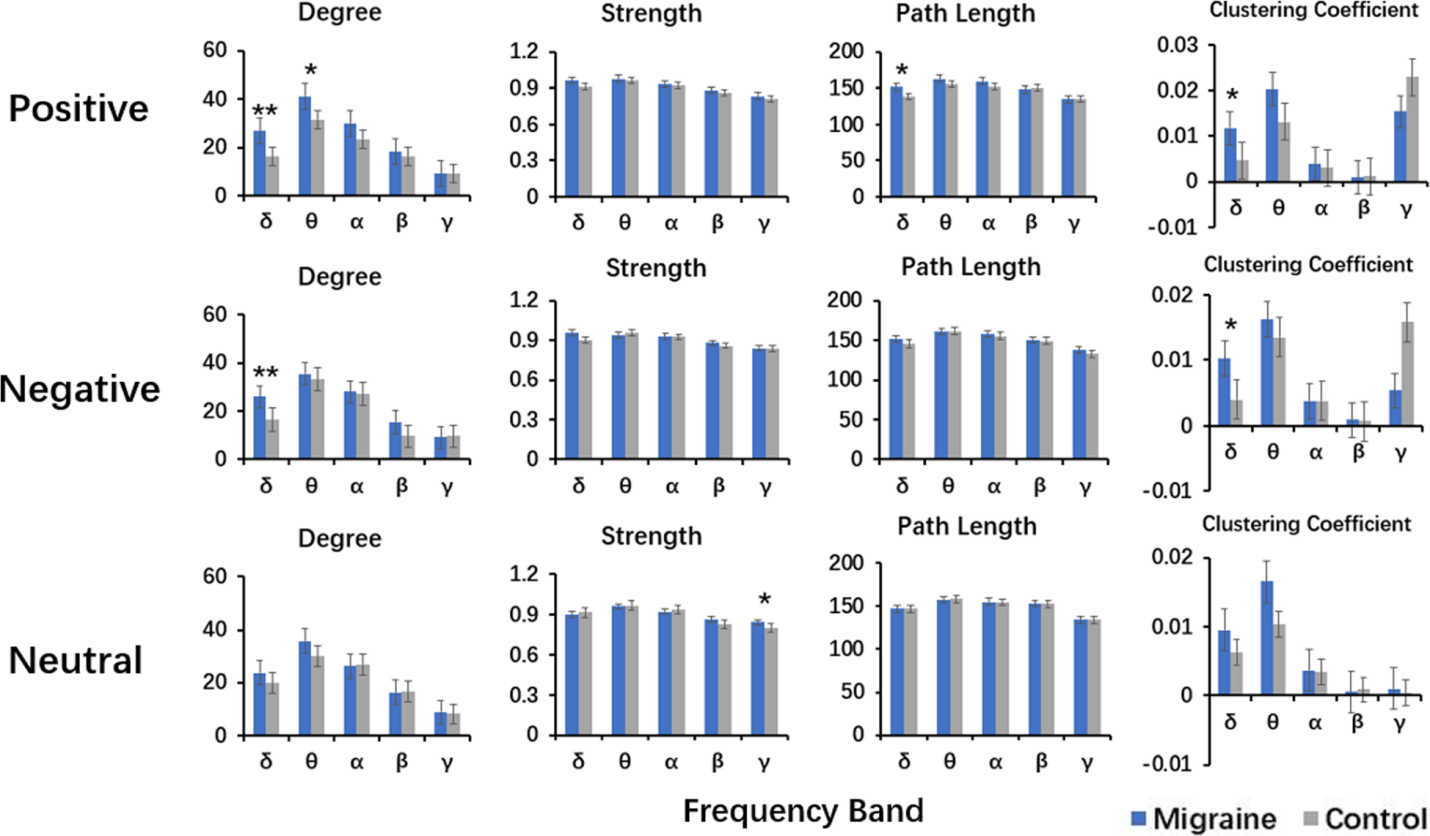
Comparison of the organization of the EC networks measured by four parameters (degree, strength, path length, and clustering coefficient) between the migraine patients and healthy controls. *p represents *P* < 0.05 before correction for multiple comparisons, **p represents *P* < 0.05 and the result was still significant after correction for multiple comparisons using the FDR controlling procedure (corrected for 5 × 4 tests) with FDR = pFDR * i/N. Here, pFDR = 0.05 (usual significance value in most statistical tests), i refers to the ranked index of the *p*-values that are computed and N to the number of tests. (Colored figure online)

In the migraine group, the significantly increased D and an increasing tendency of C were observed in the delta frequency (1–4 Hz) in viewing the positive facial expressions (*P* = 0.002 and *P* = 0.008, respectively). A tendency towards an increased L and an increased D in the delta (1–4 Hz) (*P* = 0.033) and theta (4–8 Hz) (*P* = 0.014) respectively, was also observed but the differences were not significant. In viewing the negative facial expressions, the migraine patients showed a significant increase in D (*P* = 0.001), a tendency of increasing C (*P* = 0.007) in the delta frequency (1–4 Hz). Besides a tendency towards an increased S in the gamma frequency (30-90 Hz) (*P* = 0.019), no significant difference was observed in viewing the neutral human facial expressions. The details are shown in Fig. 5.

### Clinical association

**Fig. 6:**
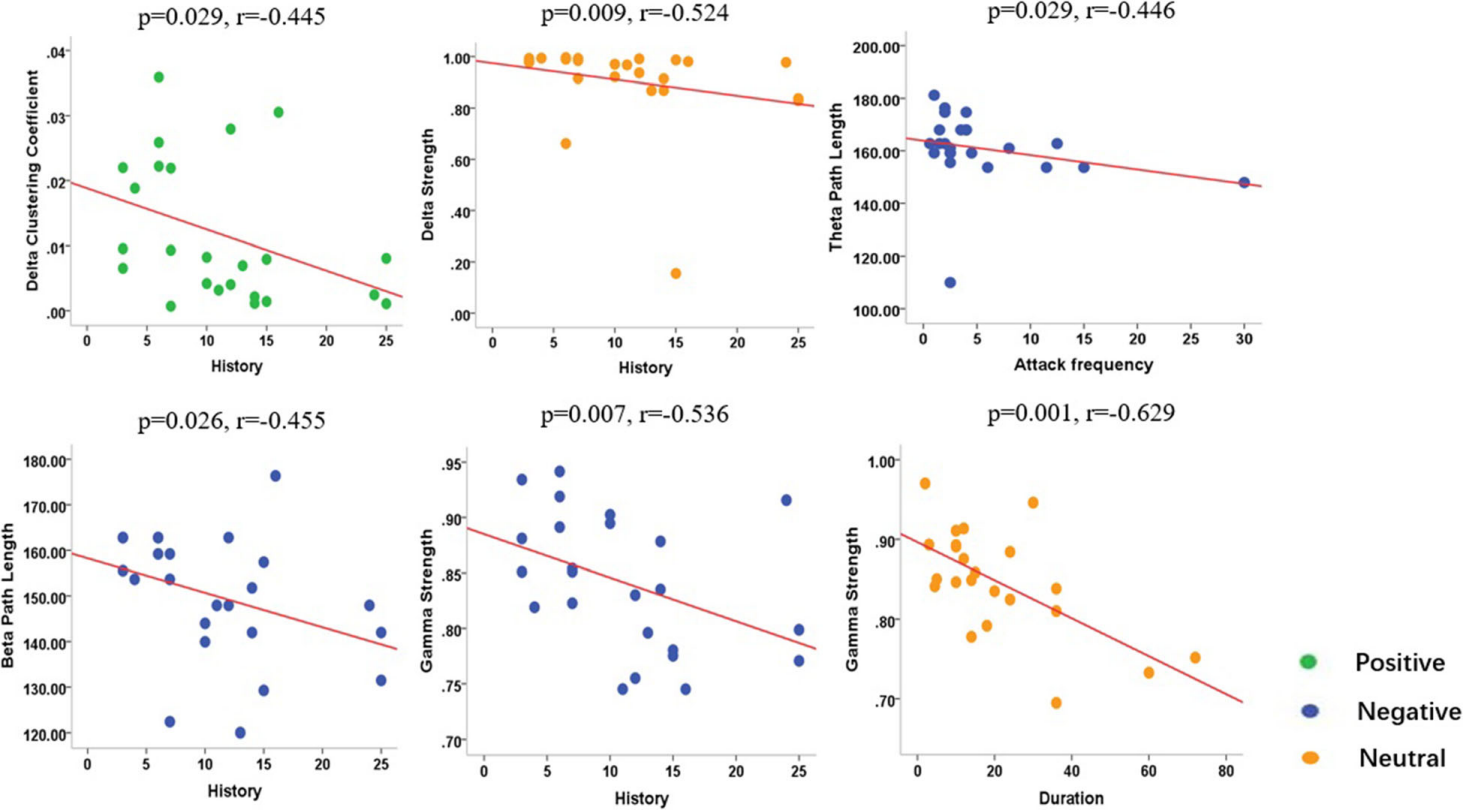
Charts of Spearman’s correlation, showing significant correlations between the parameters of network and clinical characteristics in the migraine patients in viewing the positive emotional stimuli (green point), negative emotional stimuli (blue point), and neutral stimuli (orange point). During positive emotion stimuli, the migraine history showed a negative correlation with C in the delta frequency. During negative emotion stimuli, the history of migraine showed a negative correlation with L in the beta band and with S in the gamma band, and the attack frequency of migraine also showed a negative correlation with L in the theta band. During the neutral emotion stimuli, the migraine history and duration showed a negative correlation with S in the delta and gamma frequencies, respectively

The correlations between main clinical characteristics and network patterns and parameters were analyzed. No significant correlation between the clinical features (headache history, attack frequency, and duration) and the topographic patterns of their brain network was observed (*P* >0.05). Upon exposure to the positive emotional stimuli, the migraine history showed a negative correlation with C in the delta frequency (*P* = 0.029, r = − 0.445). Upon exposure to the negative emotional stimuli, the history of migraine showed a negative correlation with L in the beta band (*P* = 0.026, r = − 0.455) and with S in the gamma band (*P* = 0.007, r = − 0.536). The attack frequency of migraine also showed a negative correlation with L in the theta (*P* = 0.029, r = − 0.446). Upon exposure to the neutral emotional stimuli, the migraine history and duration showed a negative correlation with S in the delta (*P* = 0.009, r = − 0.524) and gamma (*P* = 0.001, r = − 0.629) bands, respectively. The details are shown in Fig. 6.

**Fig. 7:**
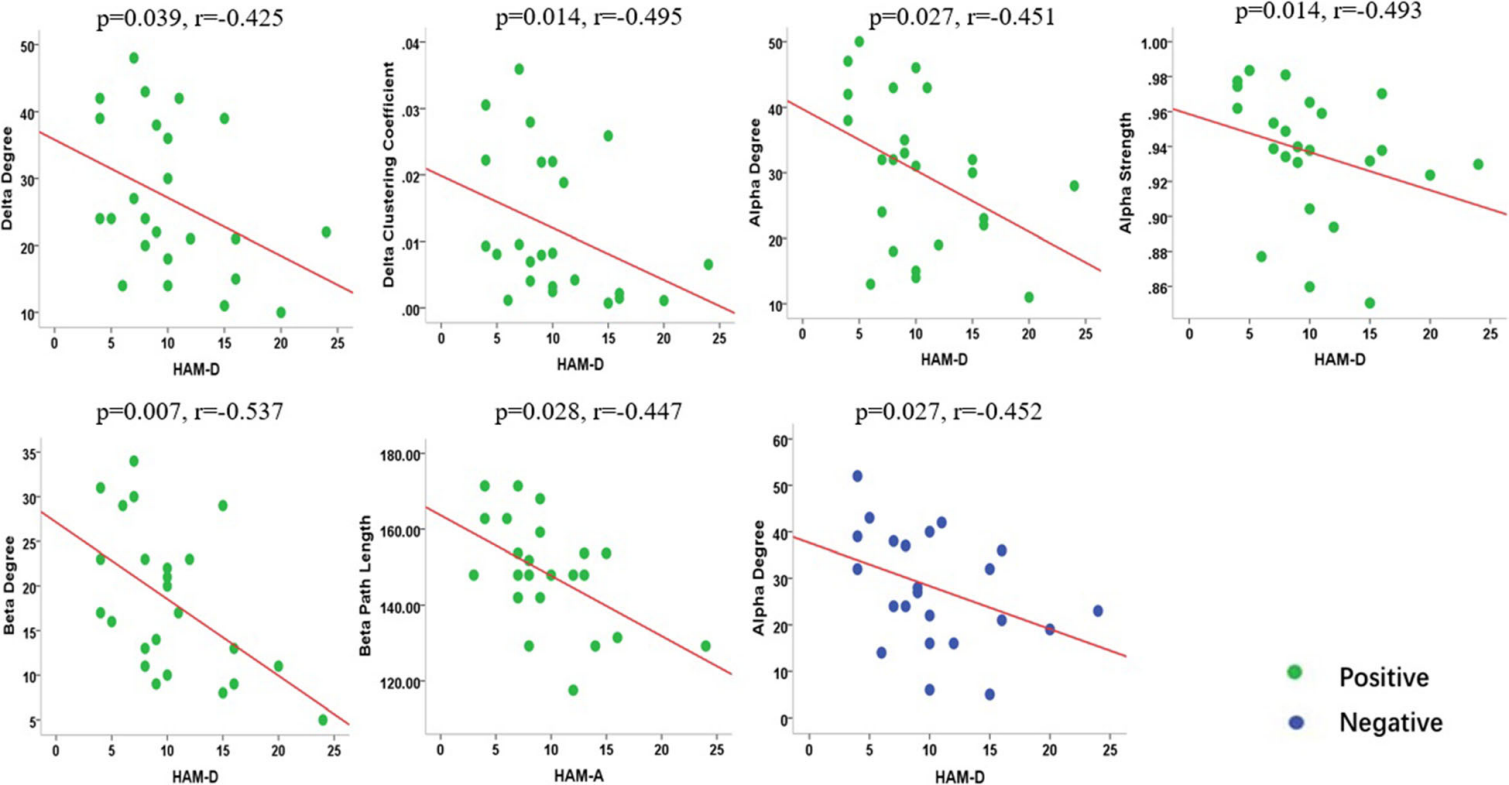
Correlation between the parameters of graph theory and neuropsychological scales in the migraine patients in viewing the positive emotional stimuli (green point) and negative emotional stimuli (blue point). In positive emotional stimuli, the HAM-D showed a negative correlation with D and C in the delta frequencies, a negative correlation with D and S in the alpha frequency as well as D in the beta frequency. HAM-A showed a negative correlation with L in the beta frequency. In negative emotional stimuli, the HAM-D score of migraine patients showed a negative correlation with D in the alpha frequency

The correlation analysis showed there was no significant correlation between the network properties and neuropsychology scales in migraine under neutral emotional stimulations. In response to positive emotional stimuli, the HAM-A and HAM-D showed a significant correlation with graph theory characteristics in some frequency ranges. In detail, HAM-D was negatively correlated with D (*P* = 0.039, r = − 0.425) and C (*P* = 0.014, r = − 0.495) in the delta frequency. Additionally, HAM-D ratings had negative correlation with D (*P* = 0.027, r = − 0.451) and S (*P* = 0.014 r = − 0.493) in alpha frequency as well as D (*P* = 0 .007, r = − 0.537) in the beta frequency. HAM-A had a negative correlation with L (*P* = 0.028, r = − 0.447) in the beta band. As for negative emotional stimuli, HAM-D score of migraine patients showed a negative correlation with D (*P* = 0.027, r = − 0.452) in the alpha frequency. No significant correlation was found between the rest of the parameters in other frequency ranges. The details are shown in Fig. 7.

## Discussion

In this study, we investigated the EC from low to high frequency among all the participants. The migraine group showed altered neural network characteristics during the headache-free phase upon exposure to stimulations by human facial expressions in distinct frequency ranges and included network patterns and brain topology measurements.

The main findings of the present study included a significant activation in migraine patients for the negative facial expression in the gamma frequency. Human facial images are important stimuli in social communications and the facial emotional expressions might be noticed in advance. This sensitivity might be related to the prioritized automatic perception of threat detection [38, 39]. In addition, de Tommaso et al. pointed that stimuli images with different emotional content could interfere with pain perception and cortical response in migraine patients [40]. Moreover, studies show that the migraine patients had abnormal neural activation in response to negative facial expressions [20, 41]. This suggested that the migraine patients might process negative emotional perceptions abnormally during the interictal phase.

Notably, the abnormal network pattern of EC was identified for the patients with migraine using the whole-brain analysis. The PFC was significantly activated in the migraine group in viewing negative pictures. As mentioned before, the fronto-temporal pathway plays a crucial role in visually presenting the emotion recognition, and these regions might sustain an over-activation through hyper-perfusion during the emotion modulation in bipolar patients [42, 43]. The PFC is mainly responsible for the attention, working memory, executive functions, and emotional regulation [44]. Consistent with this study, the PFC of migraine patients showed stronger functional connectivity with other brain areas in resting-state, which indicated that the brain regions, participating in emotion regulation, might be active even without extra emotional stimulus in the individuals with migraine [45]. A review illustrated the functional neuroimaging studies of emotional regulation in detail and proposed that PFC might act as a key part in regulating the emotion via cognitive reappraisal [46]. The comorbidity between migraine and major depressive disorder (MDD) has been widely admitted, and the dorsolateral PFC (DLPFC), as a dysfunctional region, might be correlated with the co-occurrence of these two diseases [47]. Moreover, the DLPFC was a main therapeutic target in repetitive transcranial magnetic stimulation (rTMS) for the treatment of migraine and depression [48, 49]. Combine with the previous studies, the present study suggested that the increased PFC might result from greater efforts to regulate emotion.

In addition, as compared to the healthy subjects, the migraine patients showed an enhanced activation in the temporal lobe. Previous studies have suggested that the human temporal cortex is an essential element in face and emotion perception [50]. A meta-analysis of the emotion viewing task in the healthy subjects also revealed that the bilateral fusiform and middle temporal gyrus were activated, indicating that these subregions of the temporal lobe were involved in the process of emotion [51]. Several studies have revealed structural and functional abnormalities in TL in the migraine patients, which might be related to the long-term stressful and chronic pain state in migraineurs [52, 53]. A few neuroimaging studies have proposed hyperexcitability in the temporal cortex of migraine patients in response to aversive stimuli, which was in agreement to the present study [53]. The unpleasant human facial expressions and painful heat stimuli are aversive stimuli and both can cause TL activation. Altered gray matter architecture has previously been identified in the patients with migraine [54, 55]. A structural neuroimaging study has demonstrated that the correlation temporal pole cortical thickness can be used to differentiate between the brain of a migraine patient from a healthy individual [52]. Genetic studies have pointed that the migraine and MDD are influenced by the same genes, to a certain degree [56]. Likewise, the MDD patients displayed a greater recruitment of the right middle temporal gyrus and an early increase in gamma activity at the anterior temporal region during a visual emotional task [57, 58]. The above-mentioned TL dysfunction was attributed to the unequal distribution between bottom-up hyperactive emotional processing and top-down hypoactive attention function. Taken together, the TL appeared to correspond with the capability to integrate with the emotionally related facial features.

Another major finding was that the global EC of graph theory analysis showed an altered brain network organization in the migraine patients, which indicated a significant increase in the D parameter in delta frequency under the negative and positive emotional stimulation. The tendencies towards an increased D and L were observed, although the differences were not significant. In several aforementioned MEG studies, different topological properties in the neural network of migraine patients have been demonstrated not only in the resting state but also in the specific tasks [25, 26]. D is a powerful metric to measure the centrality of a network and emphasizes the effect and significance of a brain network at the voxel level. An increase in D in the patients’ group suggested a high integration ability of a migraineur’s brain region within the cortical networks. The phenomenon of over-connectivity in migraine might be related to hyperexcitability in the brain network. C defined the possibility of neighboring nodes connections. According to a study by Watts and Strogatz, the small-world network was the most efficient network, which was characterized by highly clustered and small characteristic path length as compared to the random network and regular network [59]. The present study concluded that the patients with migraine showed an inefficient brain network in emotion-processed areas. These findings provided evidence that the long-term repeated migraine attacks might affect the emotion process in migraine patients.

The results of correlation analysis demonstrated that the parameters of graph theory in migraine patients had a negative correlation with the neuropsychological assessments and main clinical variables in the multi-frequencies after exposure to emotional stimuli. A previous study has demonstrated the duration of migraine attacks, showing a negative correlation with the network parameters of abnormal networks [26]. Besides, there was correlation between the cortical excitability and frequency of headache attacks [60]. Taken together, the history and severity of migraine might result in a more dysfunctional neural network. The migraine patients with high anxiety scores and depression scores were correlated with the high degree of migraine disability. Moreover, migraine could induce negative emotions and negative events could aggravate migraine. These findings revealed that the migraine patients showed more deficits in the processing of emotional stimuli as compared to the healthy control group, which was consistent with the conclusion of a previous study [41].

There are some limitations in the current study. The altered network pattern was only in the specific frequency range and the significant results of graph parameters were limited. The results of the present study are needed to be considered as inconclusive and hypothesis-generating. Therefore, more independent replicative studies are needed to confirm these results in future. Furthermore, several migraine patients with mild anxiety or depression from the neuropsychology scale were included in this study, which might have affected the results. Additionally, not all the migraine patients in the present study experienced ipsilateral headaches. In order to solve these problems, further studies are needed to recruit a large number of migraine patients to conduct the subgroups analysis. Moreover, the lack of a blind design was another limitation; double-blind designs in data recording and analysis should be conducted better in further studies.

## Conclusions

In summary, the present study demonstrated that the individuals with migraine showed deviant EC in viewing human facial expressions in multi-frequency ranges. The PFC pathway might be related to the altered negative emotional modulation in migraine. Furthermore, the aberrant neural network showed a negative correlation with the severity of depressive symptoms. The emotional dysregulation was possibly correlated with the pathogenesis of migraine psychiatric morbidity. Since the significance of results was for a certain frequency range, more replicative studies are needed to verify these conclusions in future. The neurocircuitry, underlying the association between migraine and emotional conditions, are needed to be elucidated in order to explain various triggers, high prevalence of comorbidity with emotional disturbances, and process of migraine chronification.

## Data Availability

The datasets used and analysed during the current study are available from the corresponding author on reasonable request.

## Abbreviations

MEG: Magnetoencephalography
EC: Effective connectivity
EEG: Electroencephalogram
fMRI: Functional magnetic resonance imaging
ICHD: International Classification of Headache Disorders
FDR: False discovery rate
VAS: Visual analog scale
HAM-A: The Hamilton Anxiety Scale
HAM-D: Hamilton Depression Scale
GC: Granger causality
D: Degree
S: Strength
L: Path length
C: Clustering coefficient
PFC: Prefrontal cortex
MDD: Major depressive disorder
DLPFC: Dorsolateral prefrontal cortex
rTMS: Repetitive Transcranial magnetic stimulation
TL: Temporal lobe
